# Combined SEP and anti-PD-L1 antibody produces a synergistic antitumor effect in B16-F10 melanoma-bearing mice

**DOI:** 10.1038/s41598-017-18641-y

**Published:** 2018-01-09

**Authors:** Zhengping Hu, Liang Ye, Yingying Xing, Jinhang Hu, Tao Xi

**Affiliations:** 1 School of Life Science and Technology, China Pharmaceutical University, Nanjing, 210009 People’s Republic of China; 2 Medicine & Pharmacy Research Center, Binzhou Medical University, Yantai, 264003 People’s Republic of China; 3 School of Public Health and Management, Binzhou Medical University, Yantai, Shandong 264003 People’s Republic of China

## Abstract

The increased PD-L1 induces poorer prognosis in melanoma. The treatment with PD-1/PD-L1 antibodies have a low response rate. The combination immunotherapies are the encouraging drug development strategy to receive maximal therapeutic benefit. In this study, we investigated the enhanced antitumor and immunomodulatory activity of combined SEP and αPD-L1 in B16-F10 melanoma-bearing mice. The results shown that combined SEP and αPD-L1 presented significant synergistic antitumor effects, increased the frequency of CD8^+^ and CD4^+^ T cells in spleen and tumor, cytotoxic activity of CTL in spleen, and IL-2 and IFN-γ levels in splenocytes and tumor. The combination treatment also produced synergistic increase in P-ERK1/2 level in spleen. Immunohistochemistry shown that SEP induced the PD-L1 expression in melanoma tissue possibly by promoting IFN-γ excretion, which led to the synergistic anti-tumor effects of aPD-L1 and SEP. Furthermore, in the purified T lymphocyte from the naive mice, the combination of SEP and αPD-L1 had more potent than SEP or αPD-L1 in promoting T lymphocyte proliferation and cytokines secretion including IL-2 and IFN-γ, at least partially by activating MEK/ERK pathway. Our study provides the scientific basis for a clinical trial that would involve combination of anti-PD-L1 mAb and SEP for sustained melanoma control.

## Introduction

Melanoma comprises only 5% of all skin cancers, but approximately 80% of all skin cancer-related deaths are caused by melanoma^1^. The average survival time was 6–12 months and a 5-year survival rate under 10% with traditional therapies^2^. Recently, due to the profound understanding of immunobiology for melanoma development, immunotherapies have become the standard treatment regimens for the patients with advanced melanoma^2^.

T lymphocytes play a critical role in cell-mediated immunity and cancer immunotherapy. The classic two-signal activation model includes both TCR signaling pathways and CD28/B7 costimulatory pathway^3^. However, the coinhibitory receptors such as anti-cytotoxic T-lymphocyte antigen-4 (CTLA-4) and programmed cell death 1 (PD-1) are able to down-regulate the immune system by preventing T cell over-activation, promoting self-tolerance and avoiding autoimmunity^4^. After expressed approximately 48 h after T cell activation, CTLA-4 binds to B7-1 in limiting T cell activation^2^. When the activated T cells enter tumor microenvironment, they become “tolerated” (functionally inactivated) by engagement of PD-1/B7-H1 (PD-L1) signaling pathway^5^. Therefore, these negative regulation mechanisms decrease T cell anti-tumor activity in cancer immunotherapy. So far, the immunotherapeutic drugs such as CTLA-4 antibody (ipilimumab) and PD-1 antibodies (pembrolizumab and nivolumab), have been approved for the treatment of the advanced melanoma by the US Food and Drug Administration (FDA)^2^. In addition, the PD-1 receptor ligand (PD-L1) antibodies (BMS-936559 and atezolizumab) have demonstrated promising activity for treating melanoma in preclinical mouse models and clinical trials^6^. Furthermore, concurrent CTLA-4 and PD-1/PD-L1 inhibition and combination with other immunotherapeutic strategies, have been a promising approach for the melanoma patients with the increased benefits^7^. In a randomized phase III trial in treating patients with metastatic melanoma, an increased response rate and improved progression-free survival were observed with the ipilimumab-nivolumab combination when compared with ipilimumab alone^8^.

Many polysaccharides, isolated from mushrooms, fungi, yeasts and plants, have attracted more attention recently due to their immunomodulatory and anti-cancer effects^9^. Several immunoceuticals composed of polysaccharides have been used for treating cancers such as lentinan, schizophyllan and krestin^9^. These polysaccharides are be characterized by low toxicity and limited side effects^10^.

Sea urchins belong to the echinoderm phylum. Sea urchin eggs are a kind of favorite seafood for their good taste and high nutrition in China^9^. *Strongylocentrotus nudus* egg polysaccharide (SEP), a D-glucan containing an a-1,4-linked backbone and a-1,6-linked branches, was isolated and purified from *Strongylocentrotus nudus* eggs^9,11^. In our previous studies, SEP prevented the growth of both S180 and H22 hepatocellular carcinomas *in vivo* by enhancing splenocyte proliferation, CD4^+^ and CD8^+^ T cell numbers as well as cytotoxic T lymphocyte (CTL) activity, and increasing IL-2 and TNF-α secretion levels in the serum^9,12^. The studies also found that SEP regulates intracellular signaling pathway associated with splenocytes proliferation and cytokine expression^9,12^.

According to the above studies, SEP can activate T cells possibly by mediating signaling pathway in lymphatic tissues, while the PD-L1 antibodies can upragulate T-cell effector function by blocking PD-1/B7-H1 (PD-L1) signaling pathway in peripheral tissues including the tumor microenvironment. Therefore, we hypothesize that the combined treatment with SEP and anti-PD-L1 mAb produce an additive and even synergistic antitumor effect via immuneregulation in melanoma.

In the present study, we investigated the enhanced antitumor and immunomodulatory activity of combined SEP and anti-PD-L1 mAb in B16-F10 melanoma-bearing mice via analyzing *in vivo* tumor growth, T lymphocytes counts, CTL cytotoxicity and cytokines expression. We also investigated the potential effects of combination treatment on MEK/ERK signaling pathway in spleen. In addition, by using the purified T lymphocyte from the untreated mice, we studied the effects of MEK/ERK signaling pathway on the T lymphocyte proliferation and cytokines secretion induced by the combined SEP and αPD-L1 *in vitro*.

## Results

### Cytoxicity

Direct cytotoxicity of SEP and/or αPD-L1 antibody was evaluated in mice melanoma B16-F10 cells *in vitro*. As expected, neither SEP or αPD-L1 mAb alone, nor combination could inhibit B16-F10 cells growth compared with the control group (each treatment *P* > 0.05 at every concentration, n = 6) (Table 1).

**Table 1:** Direct cytotoxicity of SEP and/or αPD-L1 antibody.

Gourp	Concentration (μg/mL)	48 h	72 h	96 h
SEP	1	0.92 ± 0.07	0.90 ± 0.03	0.94 ± 0.01
	3	0.97 ± 0.03	0.92 ± 0.05	0.92 ± 0.04
	10	0.95 ± 0.06	0.91 ± 0.06	0.93 ± 0.03
αPD-L1	1	0.98 ± 0.07	0.93 ± 0.09	0.95 ± 0.07
	3	0.95 ± 0.10	0.97 ± 0.03	0.91 ± 0.03
	10	0.93 ± 0.08	0.92 ± 0.06	0.93 ± 0.05
SEP + αPD-L1	1 + 1	0.90 ± 0.02	0.91 ± 0.03	0.91 ± 0.06
	3 + 3	0.93 ± 0.04	0.93 ± 0.04	0.94 ± 0.01
	10 + 10	0.91 ± 0.10	0.90 ± 0.10	0.93 ± 0.11

Note: Mice melanoma B16F10 cells were inoculated on a 96 well plate. SEP and/or αPD-L1 antibody were administered at concentrations from 1 to 10 μg/ml. Growth rates were determined by MTT assay. Values are the mean ± SEM (n = 6).

### SEP and anti-PD-L1 mAb combination inhibited the tumor growth in B16-F10 tumor isograft model

**Figure 1:**
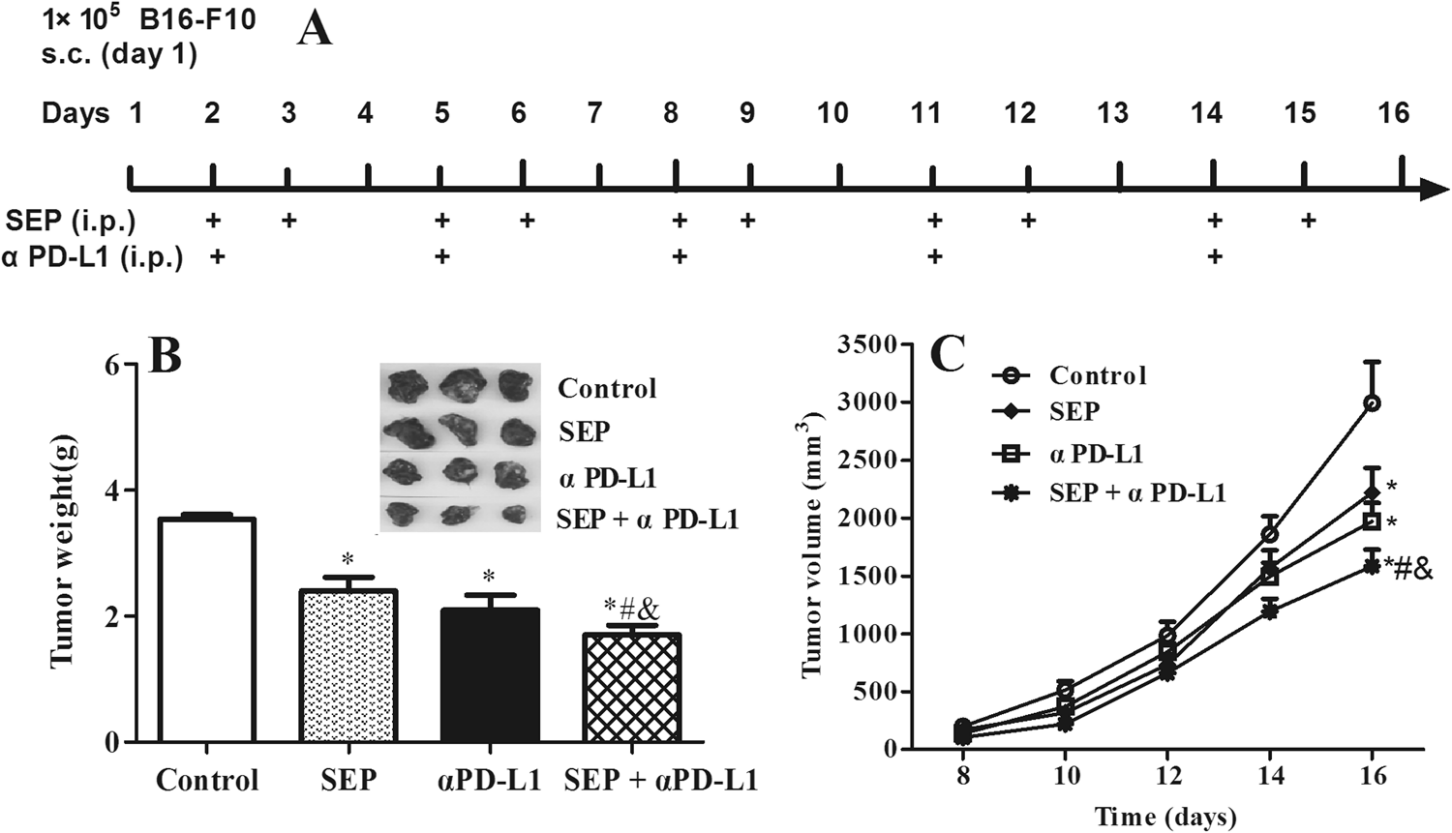
Combination of SEP and αPD-L1 results in the increased tumor growth inhibition in B16-F10 tumor isograft model. (**A**) The schema of experimental design illustrats the time points of drug administration throughout the experiment. SEP at 16 mg/kg was intraperitoneally administered on days 2, 3, 5, 6, 8, 9, 11, 12, 14 and 15, and αPD-L1 at 9 mg/kg intraperitoneally on days 2, 5, 8, 11 and 14. Tumor volumes were measured on days 8, 10, 12, 14 and 16. On day 16, all the mice were decapitated between 9:00 a.m. and 11:00 a.m. The tumors were obtained. (**B**) Combination of SEP and αPD-L1 had significantly additive tumor inhibition effects in tumor weight compared with SEP (*P* < 0.05, n = 12) or αPD-L (*P* < 0.05, n = 12). (**C**) Combination of SEP and αPD-L1 had additive tumor inhibition effects in tumor volume compared with SEP (*P* < 0.05, n = 12) or αPD-L1 (*P* < 0.05, n = 12) on day 16. The results were presented as mean ± SEM. ^*^ *P* < 0.05 compared with control group. ^#^ *P* < 0.05, compared with SEP group. ^&^ *P* < 0.05, compared with αPD-L1 group.

We examined the *in vivo* anti-cancer activities of SEP and αPD-L1 combination in mice bearing B16-F10 tumor isografts. We found that treatment with SEP significantly decreased tumor weight (*P* < 0.05, n = 12) and tumor volumes (day 16, *P* < 0.05, n = 12) compared with the control group (Fig. 1B,C). αPD-L1 alone also led to significant decrease of tumor weight (*P* < 0.05, n = 12) and tumor volumes (day 16, *P* < 0.05, n = 12). Furthermore, we noticed that combination of SEP with αPD-L1 showed more potent than SEP or αPD-L1 on reducing tumor weights (each *P* < 0.05, n = 12) and tumor volumes (day 16, each *P* < 0.05, n = 12).

### Assay of CTL activity in melanoma-bearing mice

**Figure 2:**
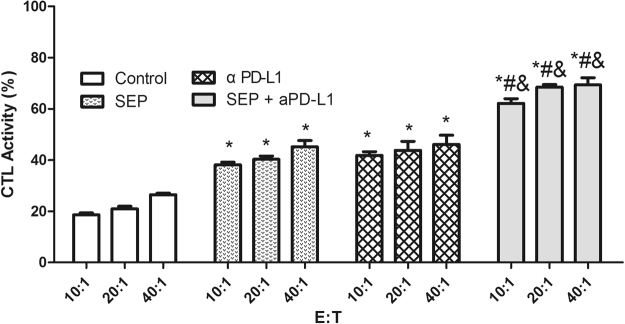
Combination of SEP and αPD-L1 results in additive upregulation of cytotoxicity activity of splenocytes in B16-F10-bearing mice. The spleens were collected from treated mice in each group (n = 3) at the end of the experiment (day 16), and the single-cell suspensions were prepared. The cytotoxic activity of splenocytes at E/T ratios of 10:1, 20:1 and 40:1 were increased in three treatment groups (each treatment *P* < 0.05 at every ratio, n = 3). Combination of SEP and αPD-L1 had significantly additive upregulation of cytotoxicity effects of splenocytes compared with SEP (*P* < 0.05 at every ratio, n = 3) or αPD-L1 (*P* < 0.05 at every ratio, n = 3). The results were presented as mean ± SEM. ^*^ *P* < 0.05 compared with control group. ^#^ *P* < 0.05, compared with SEP group. ^&^ *P* < 0.05, compared with αPD-L1 group.

CTL plays an important role in inhibiting tumor cell. Therefore, we measured the cytotoxic activity of splenocytes against B16-F10 cells. The results showed that the cytotoxic activity of splenocytes at E/T ratios of 10:1, 20:1 and 40:1 were increased in three treatment groups in B16-F10-bearing mice compared with the control group (Fig. 2). The cytotoxic activities in SEP or αPD-L1-treated mice were significantly higher than those of the corresponding control (each treatment *P* < 0.05 at every ratio, n = 3). Furthermore, combination of SEP and αPD-L1 resulted in the additive upregulation of cytotoxicity activity of splenocytes in B16-F10-bearing mice compared with SEP (*P* < 0.05 at every ratio, n = 3) or αPD-L1 (*P* < 0.05 at every ratio, n = 3).

### Flow cytometry analyses for CD3^+^, CD4^+^ and CD8^+^ T lymphocytes in tumor and spleen

**Figure 3:**
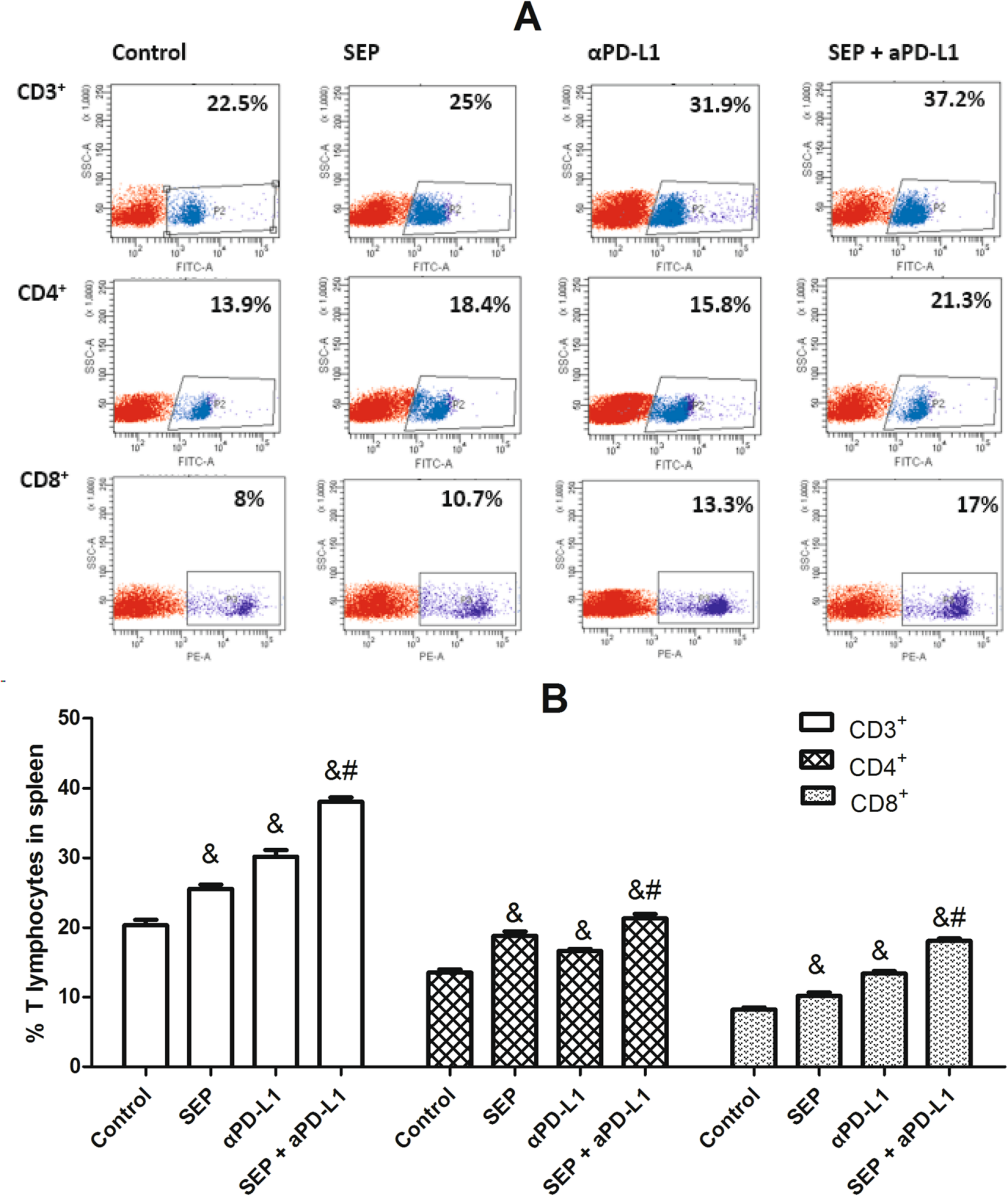
Combination of SEP and αPD-L1 on T cell subsets from spleen in B16-F10-bearing mice. The mouse spleens were harvested at the end of the experiment (day 16). The cell counts for CD3^+^, CD4^+^ and CD8^+^ T cells from mouse spleen were determined by flow cytometry. The increase in the percentage of either CD3^+^, CD4^+^ or CD8^+^ T cells was observed in three treatment groups (each treatment *P* < 0.05 for each T cell, respectively, n = 6). And, combination of SEP and αPD-L1 had significantly additive increase of either CD3^+^, CD4^+^ or CD8^+^ T cells compared with SEP (each T cell *P* < 0.05, respectively, n = 6) or αPD-L1 (each *T* cell *P* < 0.05, respectively, n = 6). The results were presented as mean ± SEM. ^*^ *P* < 0.05 compared with control group. ^#^ *P* < 0.05, compared with SEP group. ^&^ *P* < 0.05, compared with αPD-L1 group.

To investigate the effect of each treatment on the cellular immunity, the cell counts for CD3^+^, CD4^+^ and CD8^+^ T cells in tumor and spleen were determined by flow cytometry. As shown in Fig. 3, the increase in the percentage of CD3^+^, CD4^+^ or CD8^+^ T cells was observed in spleen in three treatment groups compared with the control group (each treatment *P* < 0.05 for each T cell, respectively, n = 6). Furthermore, the combination of SEP and αPD-L1 led to significantly additive increase of CD3^+^, CD4^+^ or CD8^+^ T cells in spleen compared with SEP (each T cell *P* < 0.05, respectively, n = 6) or αPD-L1 (each T cell *P* < 0.05, respectively, n = 6).

**Figure 4:**
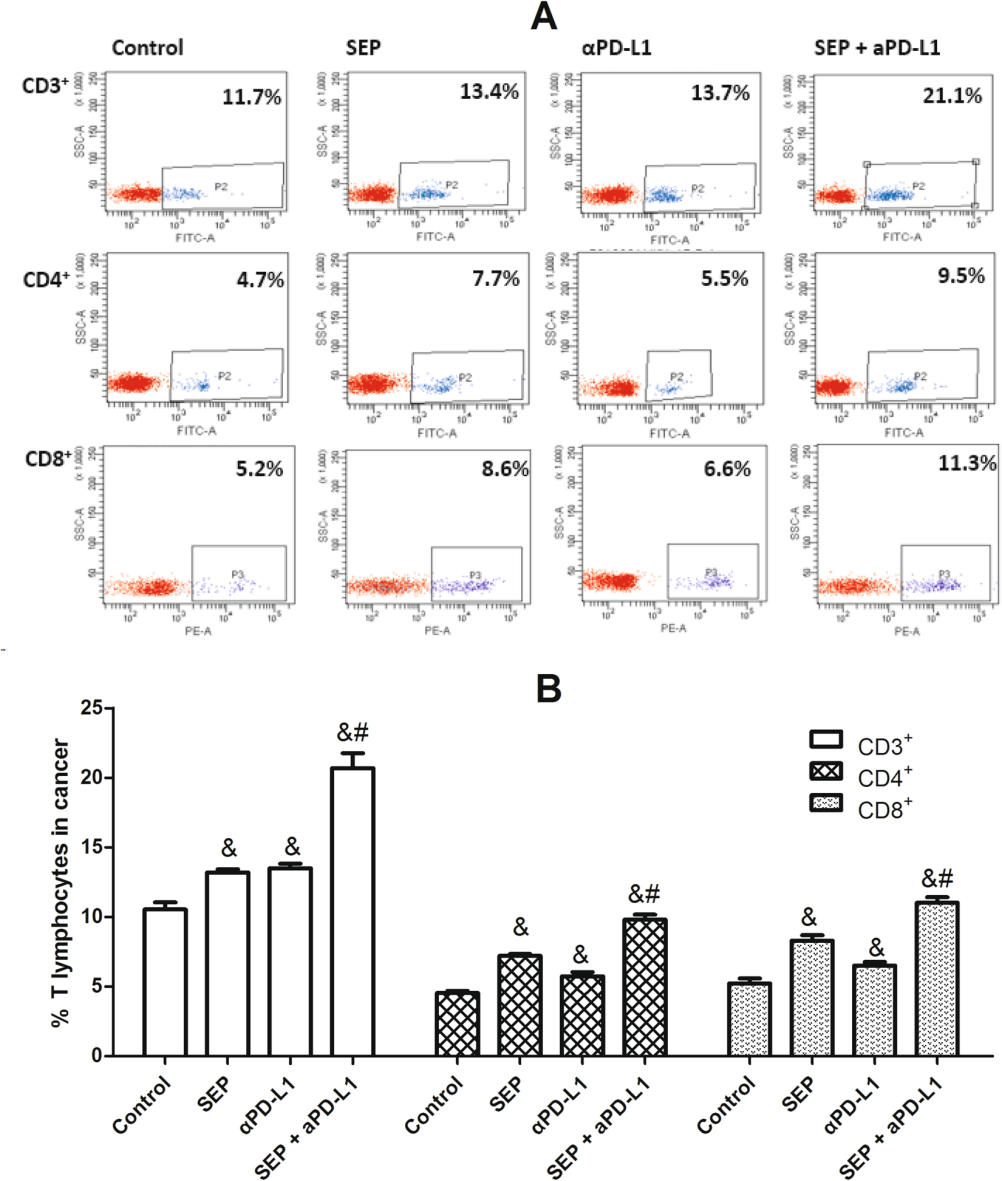
Combination of SEP and αPD-L1 on T cell subsets from tumor in B16-F10-bearing mice. The mouse tumors were harvested at the end of the experiment (day 16). The cell counts for CD3^+^, CD4^+^ and CD8^+^ T cells from mouse tumor were determined by flow cytometry. The increase in the percentage of either CD3^+^, CD4^+^ or CD8^+^ T cells was observed in three treatment groups (each treatment *P* < 0.05 for each T cell, respectively, n = 6). And, combination of SEP and αPD-L1 had significantly additive increase of either CD3^+^, CD4^+^ or CD8^+^ T cells compared with SEP (each T cell *P* < 0.05, respectively, n = 6) or αPD-L1 (each T cell *P* < 0.05, respectively, n = 6). The results were presented as mean ± SEM. ^*^ *P* < 0.05 compared with control group. ^#^ *P* < 0.05, compared with SEP group. ^&^ *P* < 0.05, compared with αPD-L1 group.

Similarly, in tumor, the increased percentage of CD3^+^, CD4^+^ or CD8^+^ T cells was also observed in three treatment groups compared with the control group (each treatment *P* < 0.05 for each T cell, respectively, n = 6) (Fig. 4). Furthermore, the combination of SEP and αPD-L1 induced significantly additive increase of CD3^+^, CD4^+^ or CD8^+^ T cells in tumor compared with SEP (each T cell *P* < 0.05, respectively, n = 6) or αPD-L1 (each T cell *P* < 0.05, respectively, n = 6).

### Tumor and spleen IFN-γ and IL-2 expression in melanoma-bearing mice

**Figure 5:**
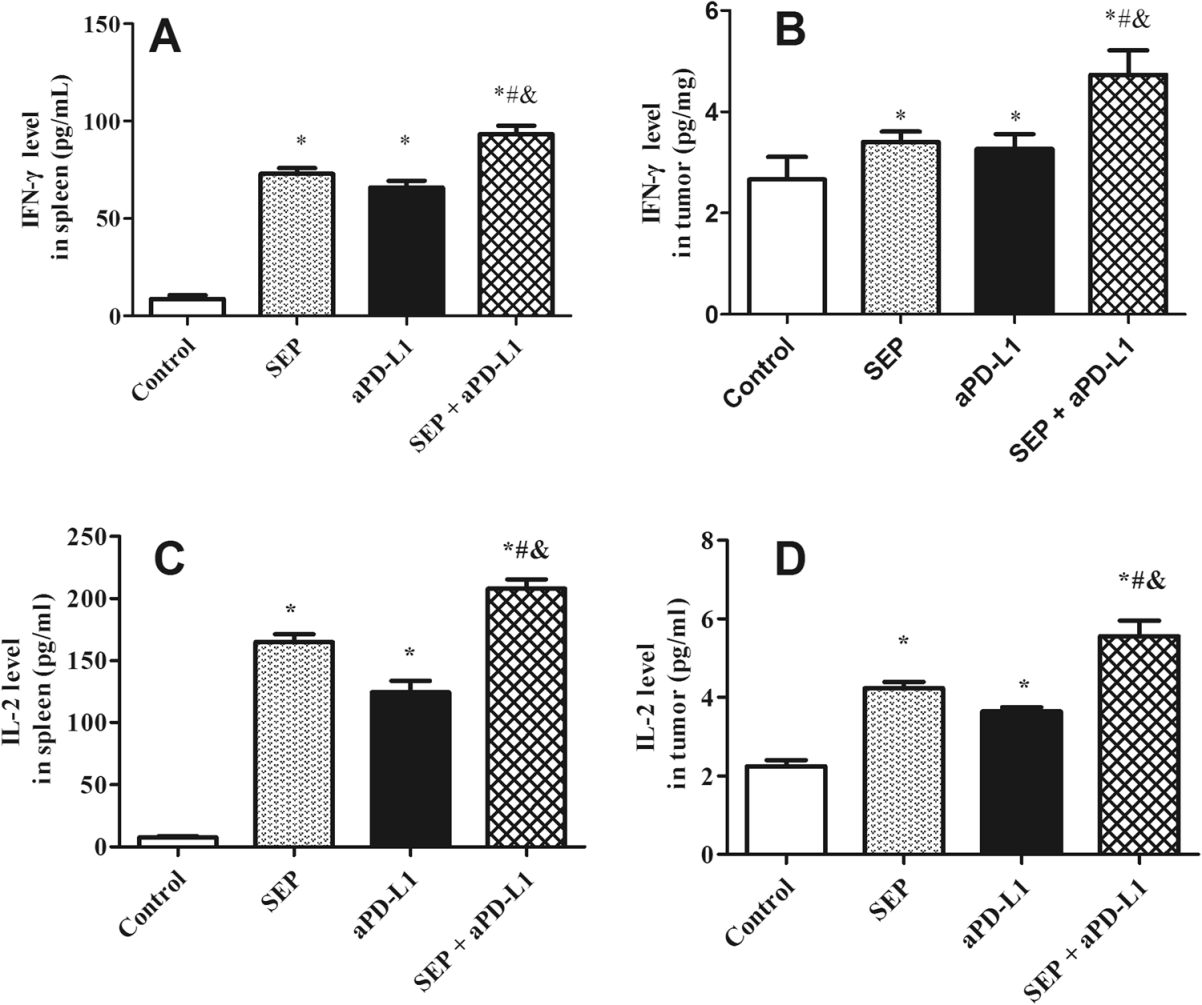
Combination of SEP and αPD-L1 on the effects of IFN-γ and IL-2 levels from spleen (**A** and **C**) and tumor (**B** and **D**) in B16-F10-bearing mice. The mouse spleens and tumors were harvested at the end of the experiment (day 16). The increase in IFN-γ and IL-2 levels in spleen or tumor was observed in three treatment groups compared with the control group (each treatment *P* < 0.05 for each cytokine, respectively, n = 6). And, combination of SEP and αPD-L1 had significantly additive increase of IFN-γ and IL-2 levels compared with SEP (each cytokine *P* < 0.05 in spleen or tumor, respectively, n = 6) or αPD-L1 (each cytokine *P* < 0.05 in spleen or tumor, respectively, n = 6). The results were presented as mean ± SEM. ^*^ *P* < 0.05, compared with control group. ^#^ *P* < 0.05, compared with SEP group. ^&^ *P* < 0.05, compared with αPD-L1 group.

Cytokines play an important role in the immune response, we investigated the effect of the combination of SEP and αPD-L1 on the production of cytokines IFN-γ and IL-2 in spleen and tumor of B16-F10-bearing mice using ELISA kits. As shown in Fig. 5, SEP or αPD-L1 markedly elevated IFN-γ and IL-2 levels in mouse spleen compared with the control group (each treatment *P* < 0.05 for each cytokine, respectively, n = 6). Furthermore, combination of SEP and αPD-L1 promoted significantly additive increase of IFN-γ and IL-2 levels compared with SEP (each cytokine *P* < 0.05, respectively, n = 6) or αPD-L1 (each cytokine *P* < 0.05, respectively, n = 6) in spleen (Fig. 5A,C).

Similarly, in tumor in B16-F10-bearing mice, SEP or αPD-L1 significantly increased IFN-γ and IL-2 levels compared with the control group (each treatment *P* < 0.05 for each cytokine, respectively, n = 6). Furthermore, combination of SEP and αPD-L1 significantly increase IFN-γ and IL-2 levels compared with SEP (each cytokine *P* < 0.05, respectively, n = 6) or αPD-L1 (each cytokine *P* < 0.05, respectively, n = 6) in tumor (Fig. 5B,D).

### Effects on MEK/ERK signaling pathway in spleen in melanoma-bearing mice

**Figure 6:**
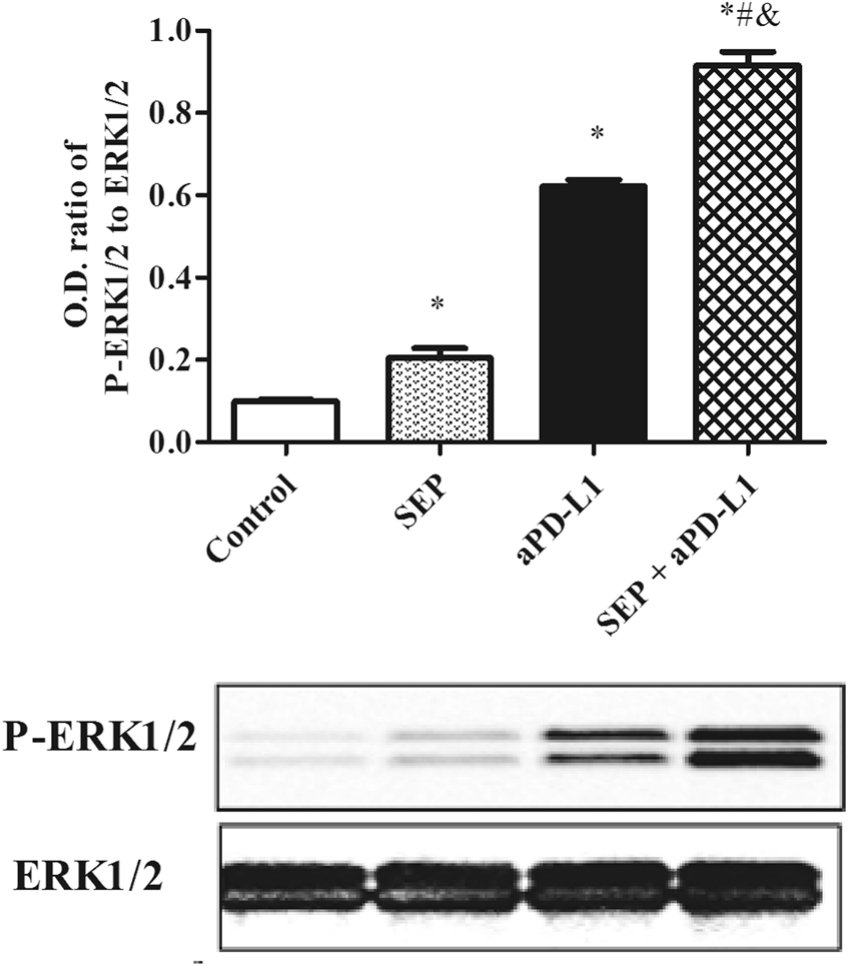
Combination of SEP and αPD-L1 on the effects of ERK1/2 phosphorylation in the spleen in B16-F10-bearing mice. The mouse spleens were harvested at the end of the experiment (day 16). The increase of P-ERK1/2 level in spleen was observed in three treatment groups compared with the control group (each treatment *P* < 0.05, respectively, n = 3). Combination of SEP and αPD-L1 significantly increases P-ERK1/2 level compared with SEP (*P* < 0.05, n = 3) or αPD-L1 (*P* < 0.05, n = 3). The results were presented as mean ± SEM. ^*^ *P* < 0.05 compared with control group. ^#^ *P* < 0.05, compared with SEP group. ^&^ *P* < 0.05, compared with αPD-L1 group.

One of the key pathways in the cytokine expression involves the MEK/ERK signaling pathway in spleen. Therefore, we investigated the effect of the combination of SEP and αPD-L1 on the ERK1/2 phosphorylation. As shown in Fig. 6, the activated ERK phosphorylation was observed in three treatment groups, compared with the control group (each treatment *P* < 0.05, n = 3). Furthermore, combination of SEP and αPD-L1 had more potent than SEP (*P* < 0.05, n = 3) or αPD-L1 (*P* < 0.05, n = 3). The results suggests that these facilitated the T cell proliferation and cytokines in B16-F10-bearing mice partially via the MEK/ERK signaling pathway.

### Immunohistochemistry

**Figure 7:**
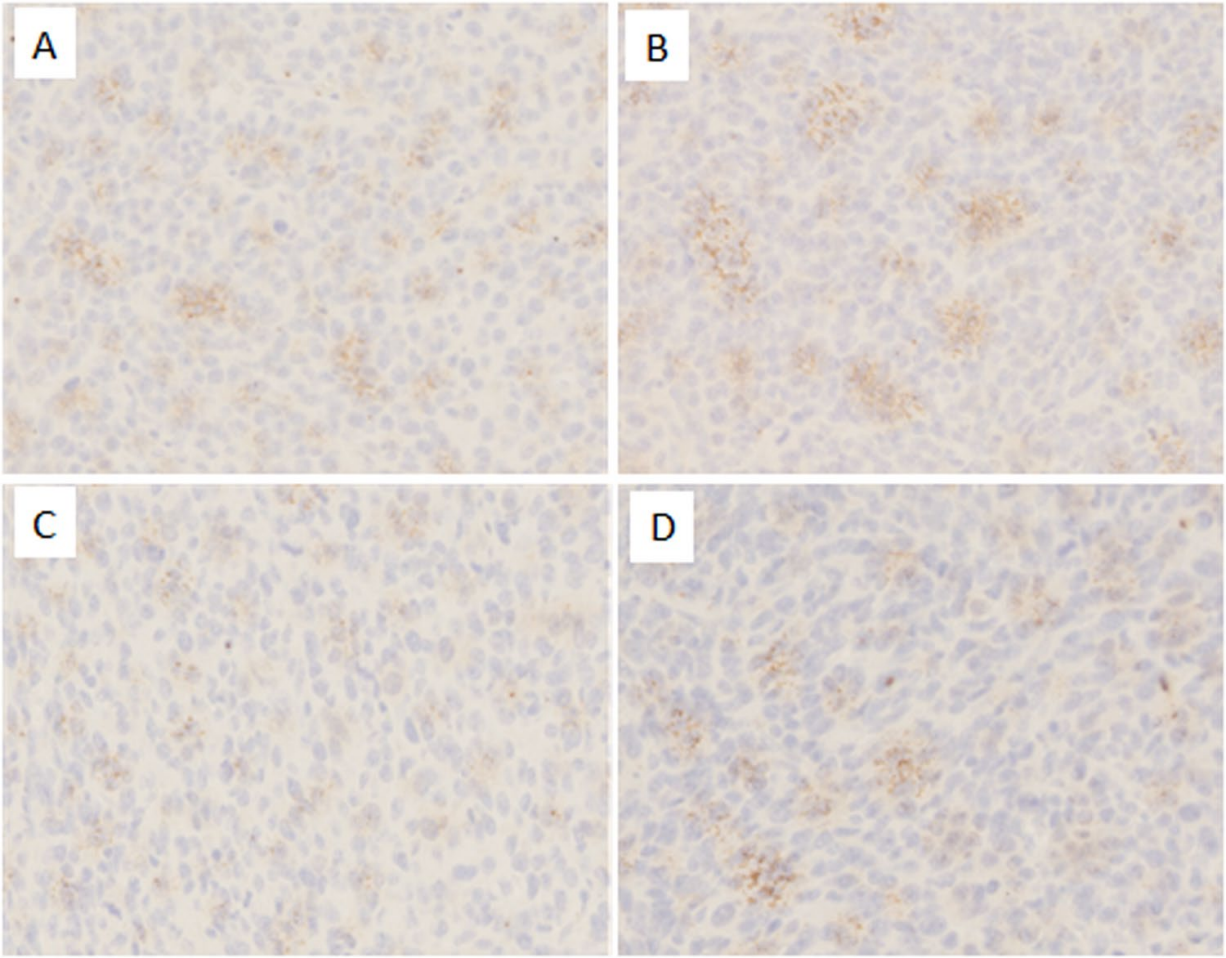
Combination of SEP and αPD-L1 on the effects of PD-L1 expression in the tumor tissue in B16-F10-bearing mice. At the end of the experiment (day 16), the collected melanoma tumors were fixed in 4% paraformaldehyde for 24 h and then embedded in paraffin. Serial sections (4-μm) were prepared for ICH. All immunostained tumor sections were examined by light microscopy at a magnification of ×100. The PD-L1 expression was significantly induced by SEP compared with that in the control group (Fig. 7A,B). The αPD-L1 decreased the PD-L1 expression compared with that in the control group (Fig. 7A,C). Furhtermore, the results in the combination group shown that αPD-L1 could decrease the PD-L1 expression induced by SEP in cancer tissue (Fig. 7B,D).

We used ICH methods to examine PD-L1 expression in melanoma cancer tissue. All immunostained tumor sections were examined by light microscopy, and images were photographed with a microscope camera (Olympus, Japan) at a magnification of × 100. We found that the PD-L1 expression was significantly induced by SEP in melanoma cancer tissue compared with that in the control group (Fig. 7A,B). The αPD-L1 decreased the PD-L1 expression compared with that in the control group (Fig. 7A,C). Furhtermore, the results in the combination group shown that αPD-L1 could decrease the PD-L1expression induced by SEP in cancer tissue (Fig. 7B,D).

### Effects of SEP and αPD-L1 combination on MEK/ERK signaling pathway in T lymphocytes *in vitro*

**Figure 8:**
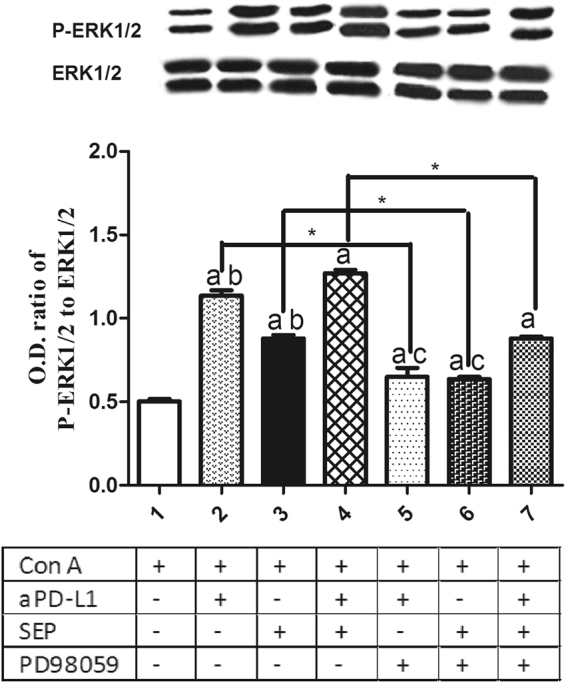
Effects of SEP and αPD-L1 combination on MEK/ERK signaling pathway in T lymphocytes *in vitro*. The T cells from naïve mice were cultured in RPMI-1640 medium supplemented with 10% fetal calf serum. After the cells were pretreated with/without MEK inhibitor PD98059 (40 μM) for 30 min, they were cultured with the aPD-L1 (10 nM) and/or SEP (50 μg/ml) with ConA (2 μg/ml) for additional 48 h. The T cells were collected and lysed in lysis buffer supplemented with a cocktail of protease and phosphatase inhibitors. Western blot analysis was conducted for the detection of P-ERK1/2 and ERK1/2 expression. αPD-L1 or SEP in combination with ConA significantly increased the ratios of P-ERK1/2 and ERK1/2 compared with ConA group (*P* < 0.05, respectively, n = 3). Furthermore, combination of SEP and αPD-L1 had more potent than SEP (*P* < 0.05, n = 3) or αPD-L1 (*P* < 0.05, n = 3). However, after the pretreatment with PD98059 for 30 min, the the increased ratios induced by SEP or/and αPD-L1 was partially decreased (*P* < 0.05, respectively, n = 3). The results were presented as mean ± SEM. ^a^ *P* < 0.05 compared with control group. ^b^ *P* < 0.05, compared with SEP and αPD-L1 combination group (group 4). ^c^ *P* < 0.05, compared with SEP and αPD-L1 combination group after PD98059 pretreatment for 30 min (group 7). ^*^ *P* < 0.05, group 2 compared with group 5; group 3 compared with group 6; group 4 compared with group 7.

To assess the role of MEK/ERK signaling pathway in T lymphocytes immunomodulation during SEP or/and αPD-L1 treatment, we used western blot analysis to detect the expression of ERK1/2 and p-ERK1/2 in T lymphocytes after 48 h exposure. Our results in Fig. 8 showed that αPD-L1 or SEP in combination with ConA significantly increased the ratios of P-ERK1/2 and ERK1/2 compared with ConA group (each *P* < 0.05, respectively, n = 3). Furthermore, combination of SEP and αPD-L1 had more potent than SEP (*P* < 0.05, n = 3) or αPD-L1 (*P* < 0.05, n = 3). However, after the pretreatment with MEK inhibitor PD98059 for 30 min, the the increased ratios induced by SEP or/and αPD-L1 was partially decreased (each *P* < 0.05, respectively, n = 3).

### Effects of MEK inhibitor on proliferation and cytokines secretion induced by SEP and αPD-L1 combination in T lymphocytes *in vitro*

**Figure 9:**
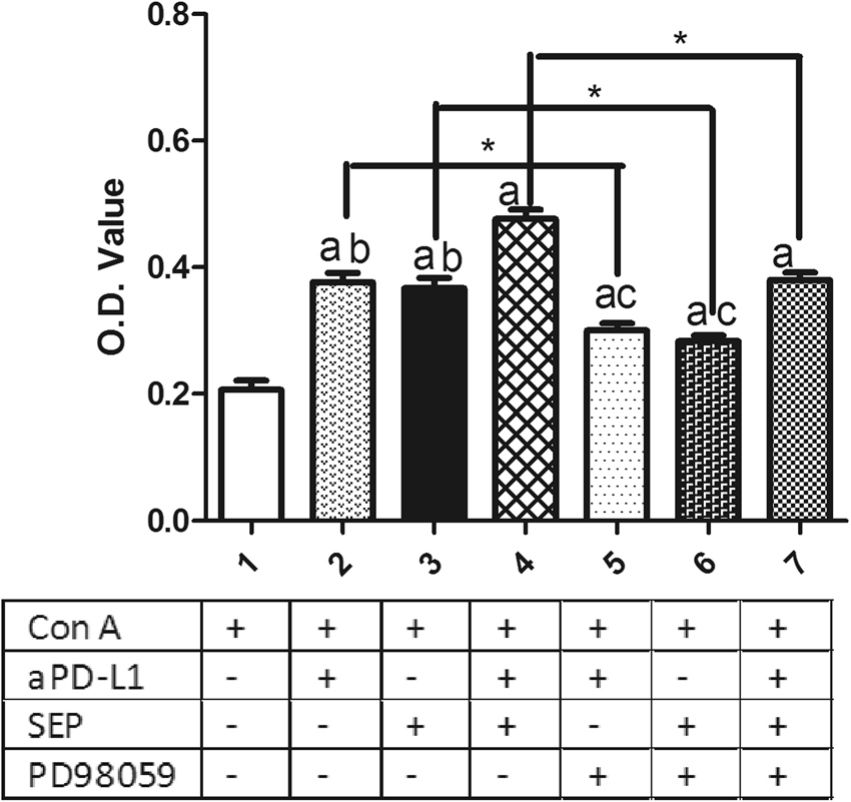
Effects of MEK inhibitor on proliferation induced by SEP and αPD-L1 combination in T lymphocytes *in vitro*. The T cells from naïve mice were cultured in RPMI-1640 medium supplemented with 10% fetal calf serum. After the cells were pretreated with/without MEK inhibitor PD98059 (40 μM) for 30 min, they were cultured with the aPD-L1 (10 nM) and/or SEP (50 μg/ml) with ConA (2 μg/ml) for additional 48 h. Then the cell survival was determined by CCK-8 method. αPD-L1 or SEP in combination with ConA significantly promoted T lymphocytes proliferation compared with ConA group (*P* < 0.05, respectively, n = 6). Furthermore, combination of SEP and αPD-L1 had more potent than SEP (*P* < 0.05, n = 6) or αPD-L1 (*P* < 0.05, n = 6). However, after the pretreatment with PD98059 for 30 min, the T cell proliferation induced by SEP or/and αPD-L1 was partially inhibited (*P* < 0.05, respectively, n = 6). The results were presented as mean ± SEM. ^a^ *P* < 0.05 compared with control group. ^b^ *P* < 0.05, compared with SEP and αPD-L1 combination group (group 4). ^c^ *P* < 0.05, compared with SEP and αPD-L1 combination group after PD98059 pretreatment for 30 min (group 7). ^*^ *P* < 0.05, group 2 compared with group 5; group 3 compared with group 6; group 4 compared with group 7.

To investigate the effects of MEK/ERK signaling in T lymphocyte proliferation, CCK-8 kit was used. As shown in Fig. 9, αPD-L1 or SEP in combination with ConA significantly promoted the T lymphocytes proliferation compared with ConA group (each *P* < 0.05, respectively, n = 6). Furthermore, combination of SEP and αPD-L1 had more potent than SEP (*P* < 0.05, n = 6) or αPD-L1 (*P* < 0.05, n = 6). However, after the pretreatment with MEK inhibitor PD98059 for 30 min, the T cell proliferation induced by SEP or/and αPD-L1 was partially inhibited (*P* < 0.05, respectively, n = 6).

**Figure 10:**
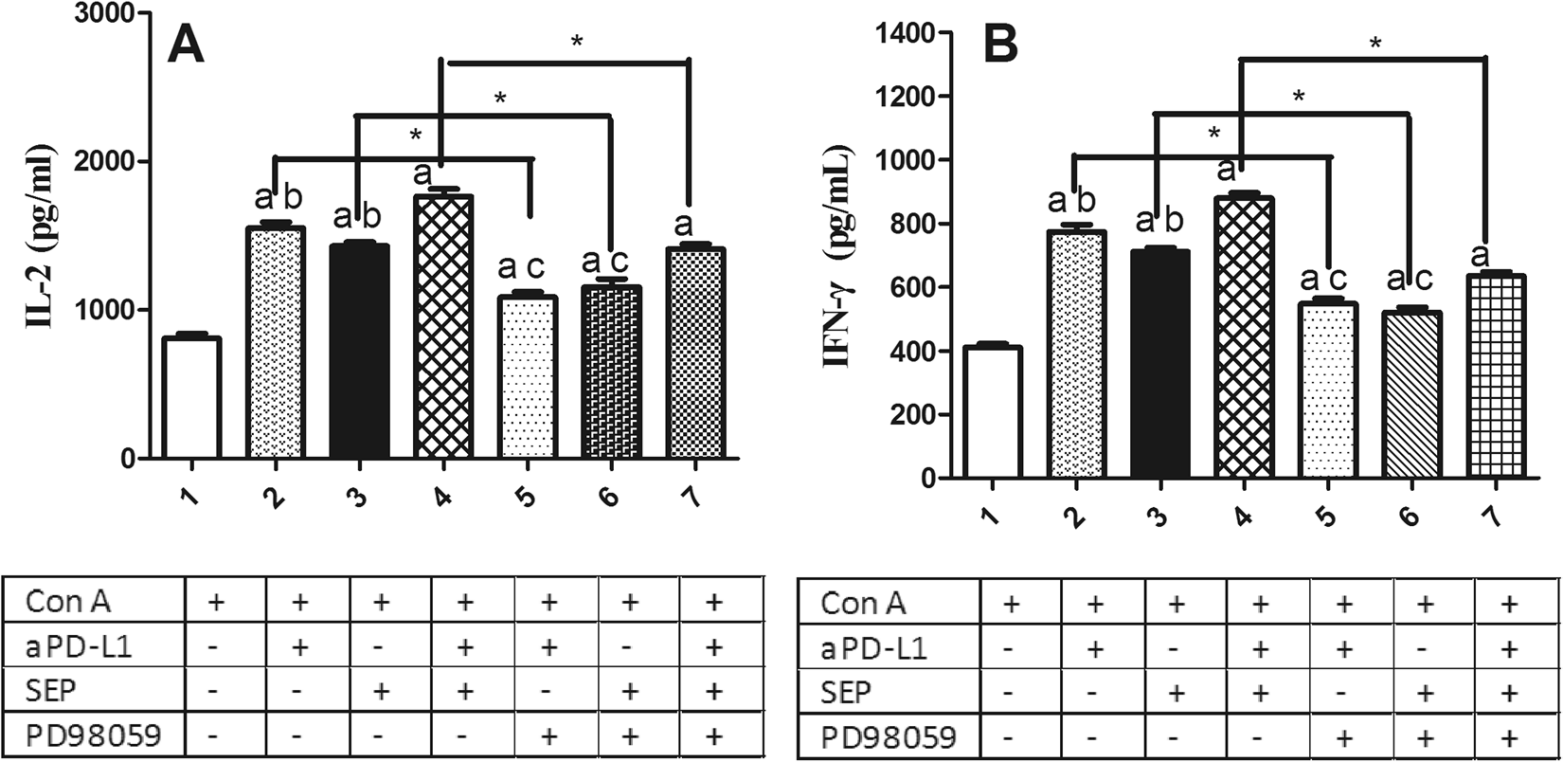
Effects of MEK inhibitor on IFN-γ and IL-2 secretion induced by SEP and αPD-L1 combination in T lymphocytes *in vitro*. The T cells from naïve mice were cultured in RPMI-1640 medium supplemented with 10% fetal calf serum. After the cells were pretreated with/without MEK inhibitor PD98059 (40 μM) for 30 min, they were cultured with the aPD-L1 (10 nM) and/or SEP (50 μg/ml) with ConA (2 μg/ml) for additional 48 h. The cell supernatants were collected, and IL-2 and IFN-γ levels were mesured by murine ELISA kits. αPD-L1 or SEP in combination with ConA significantly increased IL-2 and IFN-γ secretion (Fig. 10A,B) compared with ConA group (*P* < 0.05, respectively, n = 6). Furthermore, combination of SEP and αPD-L1 had more potent than SEP (*P* < 0.05, n = 6) or αPD-L1 (*P* < 0.05, n = 6). However, after the pretreatment with PD98059, the increased IL-2 and IFN-γ secretion in SEP or/and αPD-L1 groups was partially inhibited (Fig. 10A,B) (P < 0.05, respectively, n = 6). The results were presented as mean ± SEM. ^a^ *P* < 0.05 compared with control group. ^b^ *P* < 0.05, compared with SEP and αPD-L1 combination group (group 4). ^c^ *P* < 0.05, compared with SEP and αPD-L1 combination group after PD98059 pretreatment for 30 min (group 7). ^*^ *P* < 0.05, group 2 compared with group 5; group 3 compared with group 6; group 4 compared with group 7.

To determine the effects of MEK/ERK signaling in the regulation of IL-2 and IFN-γ secretion, we pretreated T lymphocytes with/without PD98059 for 30 min, followed by the exposure to SEP or/and αPD-L1 with ConA for 48 h. We found αPD-L1 or SEP in combination with ConA significantly increased IL-2 and IFN-γ secretion (Fig. 10A,B) compared with ConA group (*P* < 0.05, respectively, n = 6). Furthermore, combination of SEP and αPD-L1 had more potent than SEP (*P* < 0.05, n = 6) or αPD-L1 (*P* < 0.05, n = 6). However, after the pretreatment with MEK inhibitor PD98059, the increased IL-2 and IFN-γ secretion in SEP or/and αPD-L1 groups was partially inhibited (Fig. 10A,B) (P < 0.05, respectively, n = 6).

## Discussion

Melanoma has severely impacted the health and resulted in high mortality rate in all skin cancer patients. The increased PD-L1 has induced poorer prognosis in melanoma. Although the antibodies targeting PD-1/PD-L1 pathways such as pembrolizumab, nivolumab and atezolizumab can achieve long-lasting tumor regression, the low response rate still remains a major obstacle for treating melanoma patients^2,6,13^. Based on the complexity of immune regulation *in vivo*, the combination immunotherapies with different action mechanisms can receive maximal therapeutic benefit^14^ and has been the encouraging drug development strategy^15^. The ipilimumab-nivolumab combination have further increased a response rate and prolonged progression-free survival in phase III clinical trial for the melanoma patients^7,8^.

The previous studies reported that SEP has the *in vivo* antitumor activity in several animal models by increasing splenocytes proliferation and cytokines expression^9,12^. In the present study, we investigated the synergistic antitumor effect of combined SEP and anti-PD-L1 mAb in B16-F10 melanoma-bearing mice.

We found that neither SEP or α-PD-1 mAb alone, nor combination directly inhibit B16-F10 cells growth *in vitro*. However, SEP or αPD-L1 shown significant tumor growth inhibition by decreasing tumor volumes and tumor weights in B16-F10 tumor isograft model. Furthermore, combined SEP and anti-PD-L1 mAb presented significant synergistic antitumor effects. These results demonstrated that SEP and/or αPD-L1 did not produced anti-tumor effects by directly killing tumor cells. Based on the enhanced *in vivo* anti-tumor activity, we further conducted experiments to understand the possible action mechanisms of combination treatment of melanoma.

The cellular immune response by T cells plays a central role in the generation and regulation of the immune response to tumor antigens. Our study shown that less CD4^+^ and CD8^+^ T cells were found in spleen and tumor of the untreated B16-F10 tumor mice, which was associated with tumor growth. Blockade of PD1/PD-L1 signaling pathway with αPD-L1 or SEP enhanced the frequency of CD4^+^ and CD8^+^ T cells in spleen and T cells infiltration into tumor. However, the combination of SEP and αPD-L1 had synergistic effects in increasing the frequency of CD8^+^ and CD4^+^ T cells in spleen and tumor, ultimately inhibiting tumor growth. The synergistic antitumor effects with the combination of SEP and αPD-L1 were further explained by the synergistic cytotoxic activity of CTL in spleen in B16-F10 melanoma-bearing mice when compared with single treatment.

CD4^+^ T helper cells (Th) secrete a series of cytokines, such as IL-2, IFN-γ and TNF-α, to enhance the production of CD8^+^ cytotoxic T cells^9^. The IL-2 can stimulate T cell proliferation and differentiation into mature CD4^+^ and CD8^+^ T cells to enhance the adaptive immunity^16^. The activated CD8^+^ T cells can concentrate to the tumor microenvironment and secrete some cytokines, including IFN-γ. The IFN-γ expression levels by CD8^+^ T cells are closely related with the specific cytotoxic activity for antitumor effects^17^. In our study, the ELISA results showed that combined SEP and αPD-L1 induced T cells to produce the additive IL-2 and IFN-γ increase in splenocytes and tumor from B16-F10 melanoma-bearing mice when compared with single treatment, which either promote proliferation and differentiation of immune cells to inhibit tumor growth or lead to cytotoxic effects to tumor cells^9,17,18^.

We used ICH methods to examine PD-L1 expression in B16-F10 melanoma tumor tissue. The results shown that SEP significantly induced the PD-L1 expression in melanoma cancer tissue, which may partially responsible for the synergistic anti-tumor effects of aPD-L1 and SEP as tumor PD-L1 expression disabling the antitumor function of preexisting tumor antigen-specific T cells has been thought to be prerequisite for αPD-1/αPD-L1 mAb therapy to work^19^. Previous studies have demonstrated that many drugs can induce PD-L1 expression on tumor cells by increasing the IFN-γ excretion^20^. In our study, SEP can induce IFN-γ excretion in melanoma tumor tissue, which could inderectly stimulate the PD-L1 expression, which provide the rationale for combined cancer treatment of SEP and αPD-L1.

The actived PD-1/PD-L1 pathway inhibits the signaling pathways that lead to activation and expansion of T cells that recognize tumor antigens with the impaired generation of T effector and memory cells, which promote the survival of cancer cells and tumor tolerance^21^. However, blocking the PD-1/PD-L1 immune checkpoint pathway by anti-PD-1 or anti-PD-L1 antibodies, causes activation of T cells partially by increasing MEK/ERK pathway, promoting differentiation of effector and memory T cells, which suppresses cancer cell survival and enhances the antitumor responses of T cells^21^. Our previous study shown that SEP can activate MEK/ERK signaling pathway associated with T cells proliferation and cytokines excretion^9,12^. In the present study, the treatment with αPD-L1 or SEP in B16-F10 melanoma-bearing mice enhanced the ERK1/2 phosphorylation level in the spleen compared with the control group. However, the combination of SEP and αPD-L1 had synergistic effects in increasing ERK1/2 phosphorylation level in spleen, which allowed more effective T cells activation, proliferation and cytokines secretion such as IL-2 and IFN-γ, and further increase of antitumor effect.

In order to further investigate the effects of MEK/ERK signaling in T lymphocyte proliferation and cytokines secretion, we pretreated splenocytes *in vitro* from naive mice with/without MEK inhibitor PD98059 for 30 min, followed by 48 h of the coculture with the aPD-L1 and/or SEP with ConA. We found that the combination of SEP and αPD-L1 had more potent than SEP or αPD-L1 in the increase of P-ERK1/2 level. However, the pretreatment with PD98059 significantly reduced the increased P-ERK1/2 level induced by SEP or/and αPD-L1. Furthermore, SEP and αPD-L1 also significantly had more potent than SEP or αPD-L1 in promoting T cells proliferation and cytokines secretion including IFN-γ and IL-2. Similarly, the pretreatment with PD98059 significantly reduced T cells proliferation and cytokines secretion induced by SEP or/and αPD-L1. All these data indicated that the MEK/ERK signaling was at least partially responsible for T lymphocyte proliferation and cytokines secretion induced by SEP or/and αPD-L1.

The antibodies targeting PD-1/PD-L1 pathways such as pembrolizumab and nivolumab has already been approved by FDA for treatment of melanoma. The αPD-L1 has demonstrated promising activity for treating melanoma in preclinical mouse models and clinical trials. It will be critical to increase the frequency of effector T cells and cytokines excretion in order to receive maximal therapeutic benefit. Our work suggests that administration of SEP may harness the therapeutic potential of effector T cells *in vivo* and *in vitro* in combination with αPD-L1 partially by activating the MEK/ERK signaling. Our study provides the scientific basis for a clinical trial that would involve combination of αPD-L1 and SEP for sustained tumor control in cancer patients.

## Materials and Methods

### Drug and reagents

SEP was obtained from *Strongylocentrotus nudus* eggs as described previously^11^. Briefly, the crude, water-soluble polysaccharide from the eggs of sea urchins was separated and sequentially purified via Cellulose DE-52 and Sephacryl S-400 to yield the SEP for subsequent analysis. High performance liquid chromatography (HPLC) system with a TSK gel 4000 PWXL column and a Waters 2414 refractive index detector (Sigma-Aldrich, USA) was applied for analyzing the purity of SEP (mobile phase: 0.8 ml/min; column temperature: 30 °C). The profile showed a single symmetrical and sharp peak with the purity of 98.0%. To remove endotoxin contamination, 1 ml of Affi-Prep Polymyxin Matrix was packed in a Bio-spin column; centrifuged at 200 × g for 2 min; and then 0.5 ml of either SEP (100 µg/ml), LPS (2 µg/ml) or a mixture of SEP (100 µg/ml) and LPS (2 µg/ml) was added. After an overnight incubation at 4 °C, the effluent was collected and centrifuged under the same condition. The endotoxin level in SEP was determined by using the E-TOXATE kit^22^.

FITC-conjugated anti-CD3 mAb(#555274), FITC-conjugated anti-CD4 mAb (#553729) and PE-conjugated anti-CD8 mAb (#553033) were provided by BD Biosciences (California, USA). Mice interferon gamma (IFN-γ) and interleukin-2 (IL-2) ELISA kits were purchased from R&D Systems, USA. The αPD-L1 antibody (clone 10 F.9G2) was bought form Bio-XCell (USA). ERK1/2 (SC-135900), P-ERK1/2 (Thr202) (SC-101760) and lipopolysaccharide (LPS) were from Santa Cruz Biotechnology (Santa Cruz, CA, USA). The anti-PD-L1 antibody (aPD-L1, 64988S) was from Cell Signaling Technology (USA) for immunohistochemistry (ICH). PD98059 (MEK inhibitor) was purchased from Calbiochem (SanDiego, CA, USA).

### Cell lines and cell cultures

Mouse melanoma cell line B16-F10 (ATCC^®^ Number: CRL-6475™) was obtained from Cell Culture Center of the Institute of Basic Medical Sciences, Chinese Academy of Medical Sciences. The cells were maintained in DMEM supplemented with 10% (v/v) heat-inactivated fetal bovine serum in a humidified 5% CO_2_ atmosphere at 37 °C.

### Animals

Male C57BL/6NCrl mice (4–5 weeks old; purchased from Vital River Laboratory Animal Technology Co., Ltd) were used for *in vivo* experiments. Animals were maintained under controlled environment at 25 °C on a 12-h light/dark cycle, which was free access to food and water. All experiments were conducted in accordance with the guidelines of the Ministry of Health of PR China and the Animal Care Committee of China Pharmaceutical University. The study protocol was approved by the Experimental Animal Research Committee of China Pharmaceutical University.QA

### Melanoma cells growth inhibition assay

To determine the cell viability of SEP and/or aPD-L1 on melanoma cell lines, cell survival was measured by MTT assay. Cells were plated in 96-well plate with a density of 5 × 10^3^ cells/well. After 24 h incubation, the cells were exposed to SEP and/or aPD-L1 at the designated concentrations and cultured at 37 °C in a humidified atmosphere for 48 h, 72 h and 96 h. 20 μL of MTT (5 mg/ml) was added to each well and incubated for additional 4 h at 37 °C. The medium was subsequently discarded, and 150 μL of DMSO was added to dissolve the formazan crystals. The absorbance was measured at 570 nm using a Molecular Devices Spectra Max M5 (Molecular Devices, USA).

### *In vivo* tumor isograft model and dosing regimen

B16-F10 tumors were established by injecting 1 × 10^5^ cells mixed with matrigel into the dorsal area of male mice. On 2^rd^ day, the mice bearing tumors were randomly divided into four groups (12/each group). Administration doses of SEP and aPD-L1 were designed according to the references^12,14^. Mice were intraperitoneally administrated with SEP at 16 mg/kg or aPD-L1 at 9 mg/kg with a volume of 0.1 ml/10 g for each drug. The mice in combination drug group were administrated with SEP at 16 mg/kg and aPD-L1 at 9 mg/kg. Control mice were given the same volume of saline. Figure 1A showed the schema of experimental design illustrating the time points of drug administration throughout the experiment. On days 8, 10, 12, 14 and 16, body weights and tumor dimensions were measured. Tumor volumes were calculated according to the following formula: volume (mm^3^) = 0.5 × length (mm) × width (mm) × width (mm). On day 16, all the mice were decapitated between 9:00 a.m. and 11:00 a.m. The tumors were obtained. And the inhibition rate (IR) of tumor growth was calculated by the following formula: IR (%) = [(A − B)/A] × 100, where A and B were the mean tumor weight in the control and treatment groups, respectively.

### Assay of cytotoxic T lymphocyte (CTL) activity in melanoma-bearing mice

The cytotoxic activity of CTL was analyzed by MTT assay as previously described^23^. Briefly, B16-F10 cells were applied as target cells (T) and seeded in 96-well U-bottom microtiter plate at 1 × 10^4^ cells/well in DMEM complete medium. The spleens were collected from treated mice in each group (n = 3) at the end of the experiment (day 16), and gently homogenized and passed through a 200-mesh sieve to obtain single-cell suspensions. After treatment with erythrocyte lysis buffer, the splenocytes were washed with phosphate-buffered saline (PBS) three times and resuspended in DMEM complete medium, which were used as effector cells (E). The ratios of effector cells to target cells were 10:1, 20:1, and 40:1. The plates were then continuously incubated for 20 h at 37 °C. 20 µl of MTT solution (5 mg/ml) was added to each well and the plates were incubated for another 4 h. To determine the percentage of the killed target cells, the following equation was used: CTL activity (%) = (OD_T_ − (OD_S_ − OD_E_))/OD_T_ × 100%, where OD_T_, optical density value of target cells control, OD_E_, optical density value of effector cells control, and OD_S_, optical density value of samples.

### Flow cytometry analyses for CD3^+^, CD4^+^ and CD8^+^ T lymphocytes in tumor and spleen from melanoma-bearing mice

For flow cytometric analysis, spleen and tumor tissues were harvested at the end of the experiment (day 16). Single cell suspensions were prepared and a Ficoll-Hypaque purification step was carried out for the tumor-derived cell suspension. After the cells were washed twice with PBS and resuspended in 1% paraformaldehyde (PFA), about 2 × 10^6^ cells were used for antibody staining by using FITC-conjugated anti-CD3 mAb, FITC-conjugated anti-CD4 mAb, or PE-conjugated anti-CD8 mAb. The cell counts for the CD3^+^, CD4^+^ and CD8^+^ T lymphocytes were assessed via flow cytometry (BD FACSCanto II, California, USA) and expressed as a percentage of the total number of lymphocytes.

### Measurements for tumor and spleen cytokines expression in melanoma-bearing mice

Mouse splenocytes in each group were obtained as described above. After incubation with LPS (4 μg/ml) for 48 h, the supernatants were collected for detecting IFN-γ and IL-2 levels using murine ELISA kits according to the manufacturer’s instructions. And IFN-γ and IL-2 levels in the tumor supernatants were measured by ELISA kits.

### Western blot analysis

The protein content of the spleen homogenates from the melanoma-bearing mice were measured by BCA method. The protein samples (20–30 μg per lane) were separated by SDS polyacrylamide gel electrophoresis, and transferred onto a nitrocellulose membrane. These membranes were incubated with 1% bovine serum albumin in Tween-Tris-buffered saline and reacted overnight at 4 °C with the mouse or rabbits antibody (1:1000) against P-ERK1/2 and ERK1/2. After repeated washings, the immunoreactive bands were reacted with horseradish peroxidase-conjugated anti-mouse or anti-rabbit antibody, then visualized by FluorChem™ Q chemiluminescent System (ProteinSimple Corp., Canada). The O.D. value of reactive bands visible was determined densitometrically.

### Immunohistochemistry

The collected melanoma tumors were fixed in 4% paraformaldehyde for 24 h and then embedded in paraffin. Serial sections (4-μm) were prepared for ICH. After deparaffinization and antigen retrieval, the sections were preincubated in blocking serum, and then incubated overnight at 4 °C with the anti-PD-L1 antibody for ICH. Following incubation, the sections were washed three times in PBS and then incubated with biotin-conjugated secondary antibody for 2 h. Next, the sections were washed 3 times in PBS. Diaminobenzidine substrate was placed on each tissue section. After color development, the slides were immediately washed with H_2_O for 10 min, and then counterstained with hematoxylin. After dehydration by using an ethanol series and xylene, each stained section was examined by light microscopy (Olympus, Japan).

### The effects on splenocytes proliferation, cytokines expression and MEK/ERK signaling pathway *in vitro*

T cells were purified from the spleens from naive C57BL/6NCrl mice, by using magnetic negative selection (StemCell Inc., Vancouver, BC, Canada). The purity of T cells was more than 95% by as determined by flow cytometric analysis^24^. The T cells were cultured at 5 × 10^6^–6 × 10^6^ cells/ml in RPMI-1640 medium supplemented with 10% fetal calf serum. After the cells were pretreated with/without MEK inhibitor PD98059 (40 μM) for 30 min^25^, they were cultured with the aPD-L1 (10 nM) and/or SEP (50 μg/ml) with concanavalin A (ConA, 2 μg/ml) for additional 48 h. Then the cell survival was determined by CCK-8 method, or the cell supernatants were collected, and IFN-γ and IL-2 levels were mesured by murine ELISA kits.

For the effects on MEK/ERK signaling pathways, after the treatment with/wihtout PD98059 (40 μM) followed by aPD-L1 and/or SEP with ConA according to the above method, the T cells were collected and lysed in lysis buffer supplemented with a cocktail of protease and phosphatase inhibitors (Roche, Laval, QC, Canada). Western blot analysis was conducted for the detection of P-ERK1/2 and ERK1/2 expression accoriding to the above method.

### Statistical Analyses

The statistical analyses were performed using one-way ANOVA, followed by LSD post hoc test in SPSS software (V 16.0). *P* < 0.05 was considered statistically significant.
